# Supplementary material for: Uncharacterized yeast gene YBR238C, an effector of TORC1 signaling in a mitochondrial feedback loop, accelerates cellular aging via HAP4- and RMD9-dependent mechanisms
Source: eLife. 2024 May 7;12:RP92178. doi: 10.7554/eLife.92178 (PMC11076046; doi:10.7554/eLife.92178)
Supplement: Figure 2—source data 1. [file elife-92178-fig2-data1.zip › RNA seq, TF and Metascape/Metascape analysis/Enrichment_GO/ColorByCluster.pdf]

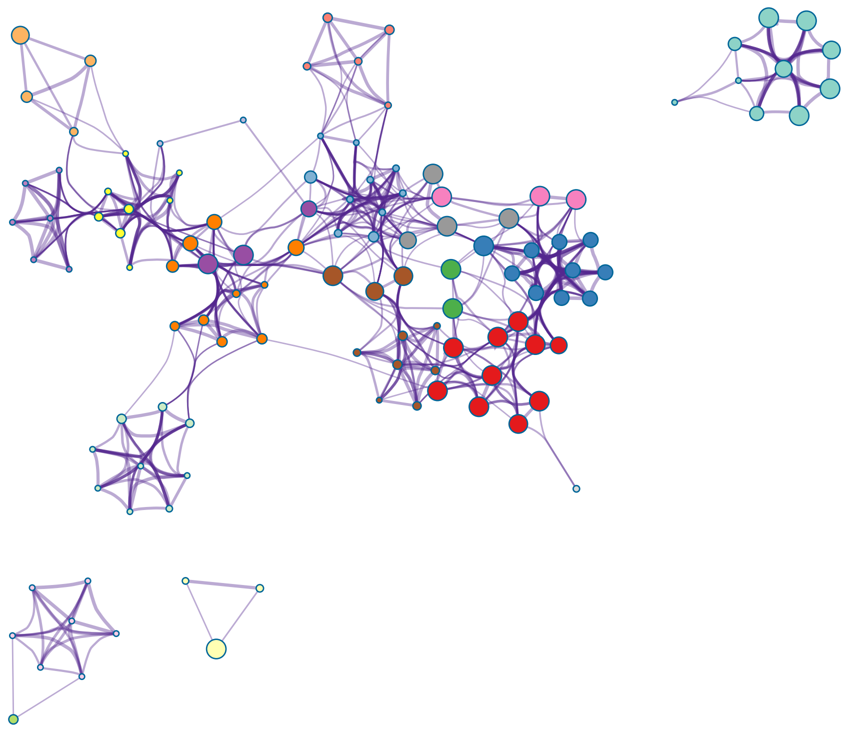

- ATP metabolic process
- ATP biosynthetic process
- Metabolic pathways - Saccharomyces cerevisiae (bu
- carbohydrate metabolic process
- Starch and sucrose metabolism - Saccharomyces cer
- trehalose metabolic process
- Principal pathways of carbon metabolism
- phosphorus metabolic process
- small molecule metabolic process
- response to extracellular stimulus
- cristae formation
- Trehalose degradation, low osmolarity
- carbohydrate transmembrane transport
- monosaccharide metabolic process
- response to heat
- Longevity regulating pathway - multiple species - Sac
- protein kinase A signaling
- mitochondrial electron transport, cytochrome c to oxy
- response to desiccation
- regulation of carbohydrate metabolic process

created by
http://metascape.org
