# Supplementary material for: Uncharacterized yeast gene YBR238C, an effector of TORC1 signaling in a mitochondrial feedback loop, accelerates cellular aging via HAP4- and RMD9-dependent mechanisms
Source: eLife. 2024 May 7;12:RP92178. doi: 10.7554/eLife.92178 (PMC11076046; doi:10.7554/eLife.92178)
Supplement: Figure 2—source data 1. [file elife-92178-fig2-data1.zip › RNA seq, TF and Metascape/Metascape analysis/Enrichment_PPI/MyList_MCODE_ALL_PPIColorByCluster.pdf]

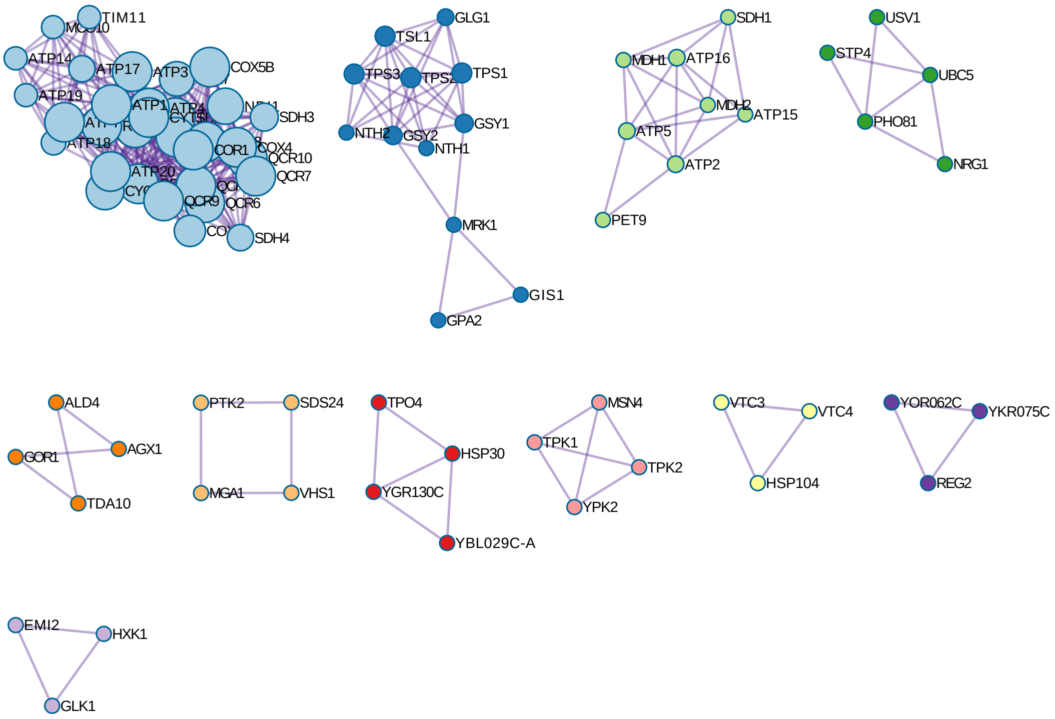

TIM11
ATP14
ATP17
ATP3
COX5B
ATP19
ATP1
ATP4
SDH3
ATP18
COR1
COX4
QCR10
ATP20
QCR7
QCR9
QCR6
SDH4
GLG1
TSL1
TPS3
TPS1
GSY1
NTH2
GSY2
NTH1
MRK1
GIS1
GPA2
SDH1
MDH1
ATP16
MDH2
ATP15
ATP5
ATP2
PET9
USV1
STP4
UBC5
PHO81
NRG1
ALD4
AGX1
GOR1
TDA10
PTK2
SDS24
MGA1
VHS1
TPO4
HSP30
YGR130C
YBL029C-A
MSN4
TPK1
TPK2
YPK2
VTC3
VTC4
HSP104
YOR062C
YKR075C
REG2
EMI2
HXK1
GLK1

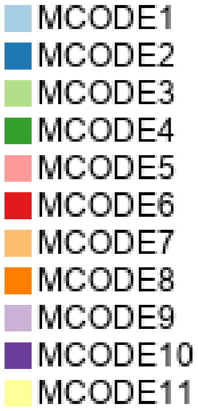

created by
http://metascape.org
