# Supplementary figures and images for: Uncharacterized yeast gene YBR238C, an effector of TORC1 signaling in a mitochondrial feedback loop, accelerates cellular aging via HAP4- and RMD9-dependent mechanisms
Source: eLife. 2024 May 7;12:RP92178. doi: 10.7554/eLife.92178 (PMC11076046; doi:10.7554/eLife.92178)

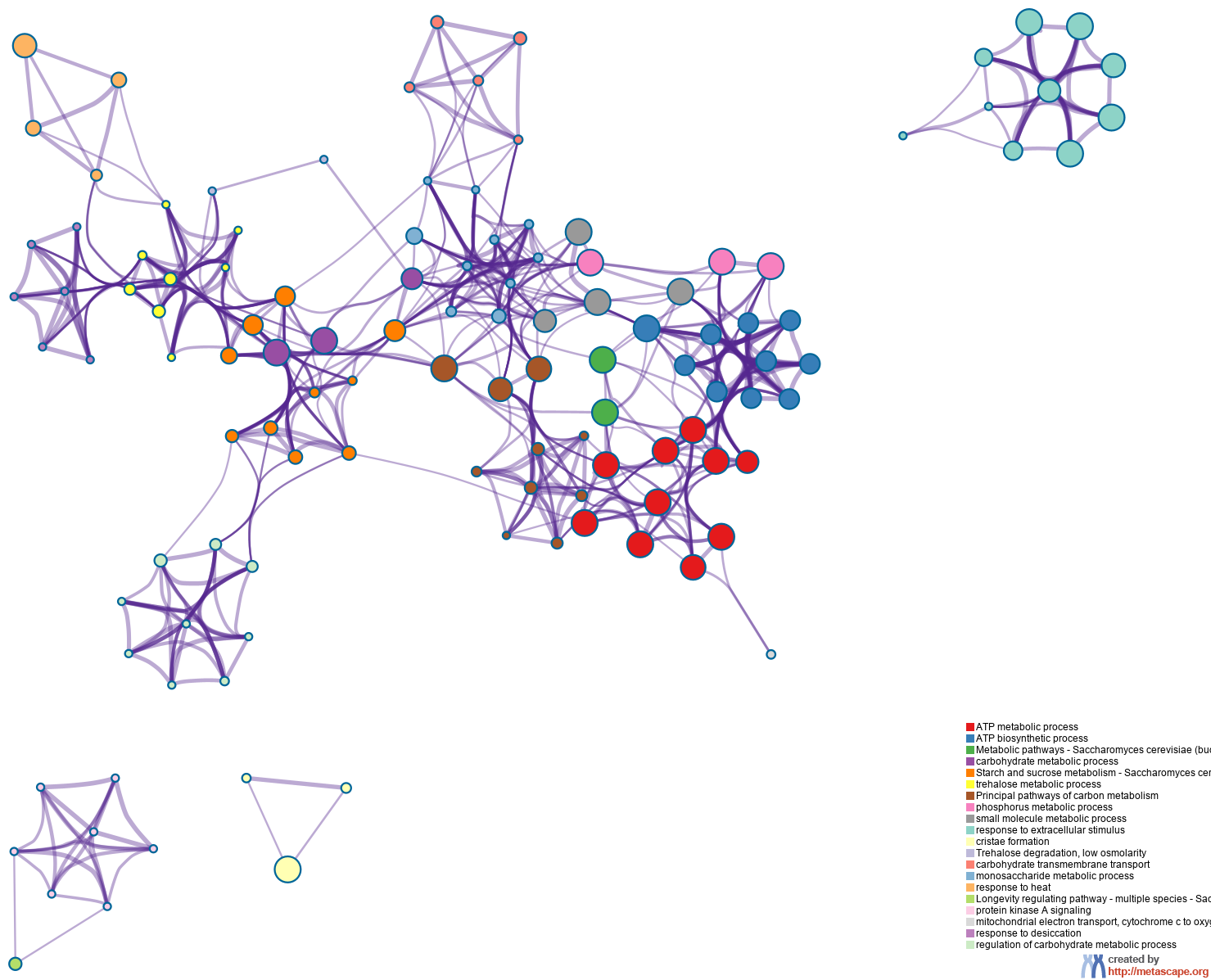

Supplement: Figure 2—source data 1. [file elife-92178-fig2-data1.zip › RNA seq, TF and Metascape/Metascape analysis/Enrichment_GO/ColorByCluster.png]

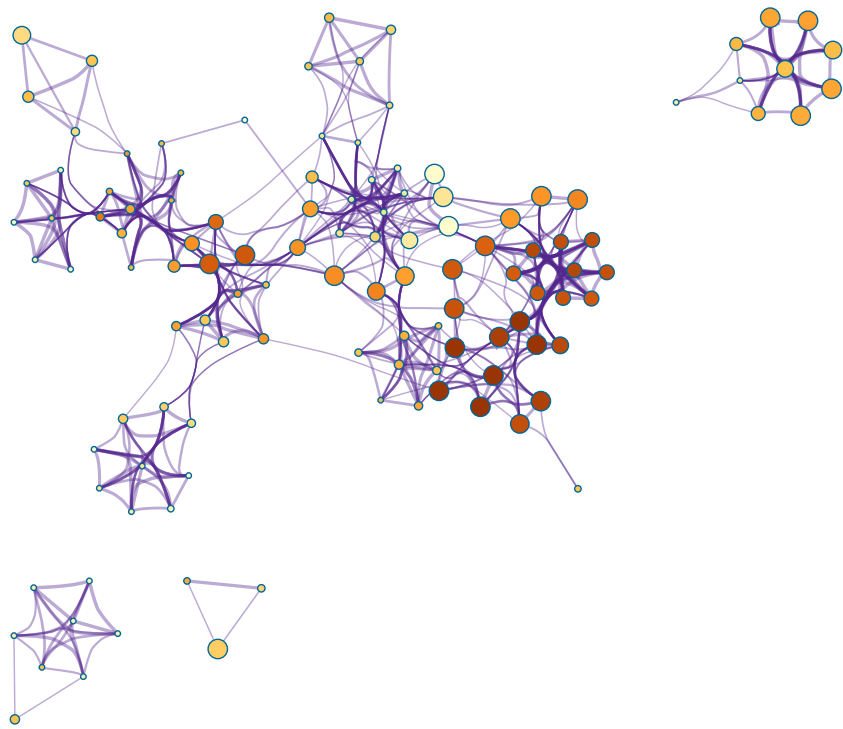

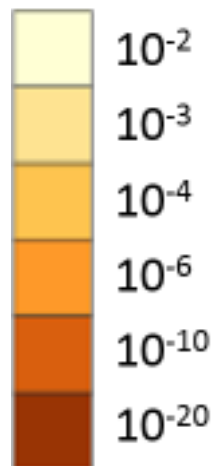

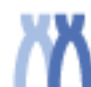 created by  
<http://metascape.org>

Supplement: Figure 2—source data 1. [file elife-92178-fig2-data1.zip › RNA seq, TF and Metascape/Metascape analysis/Enrichment_GO/ColorByPValue.pdf]

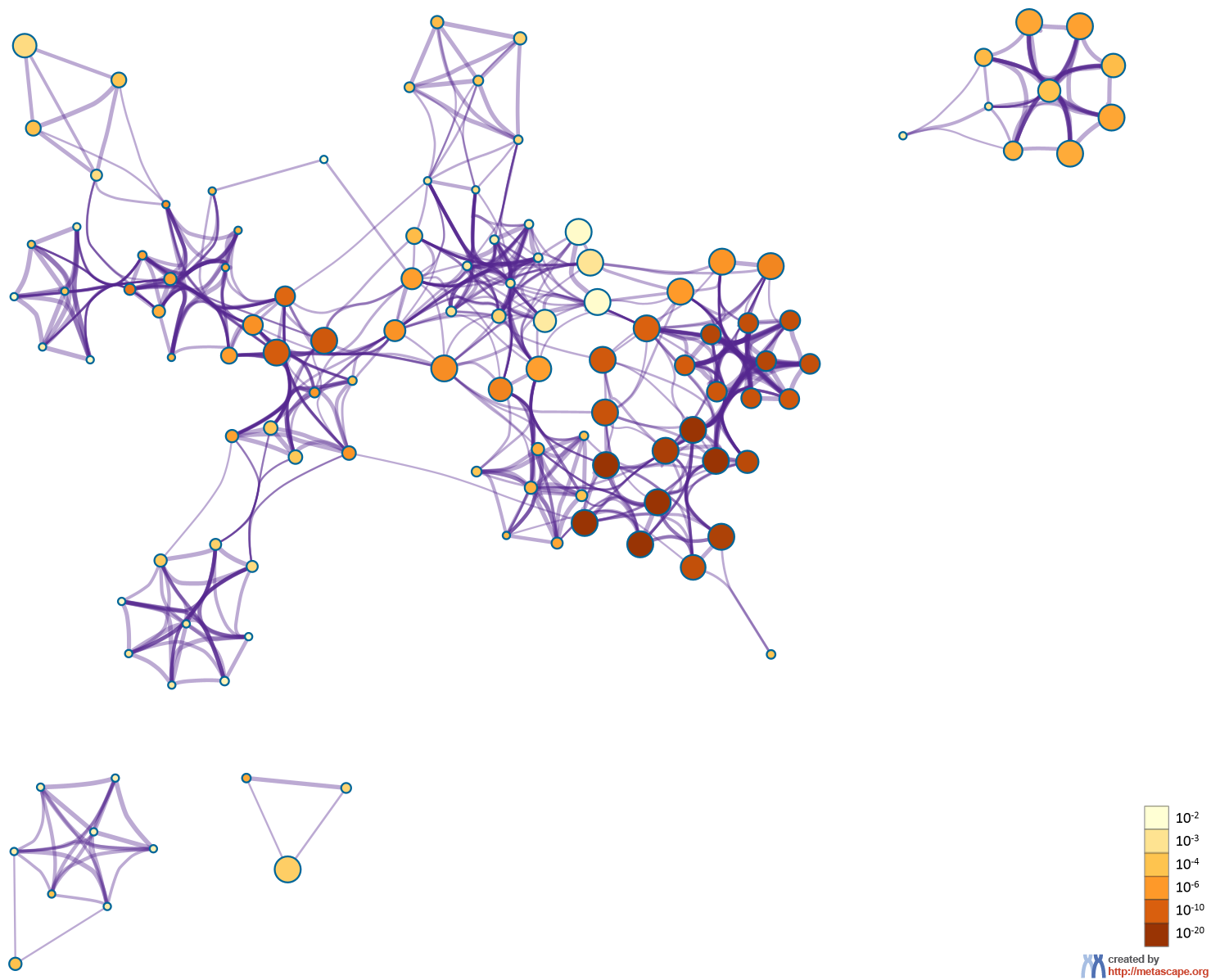

Supplement: Figure 2—source data 1. [file elife-92178-fig2-data1.zip › RNA seq, TF and Metascape/Metascape analysis/Enrichment_GO/ColorByPValue.png]

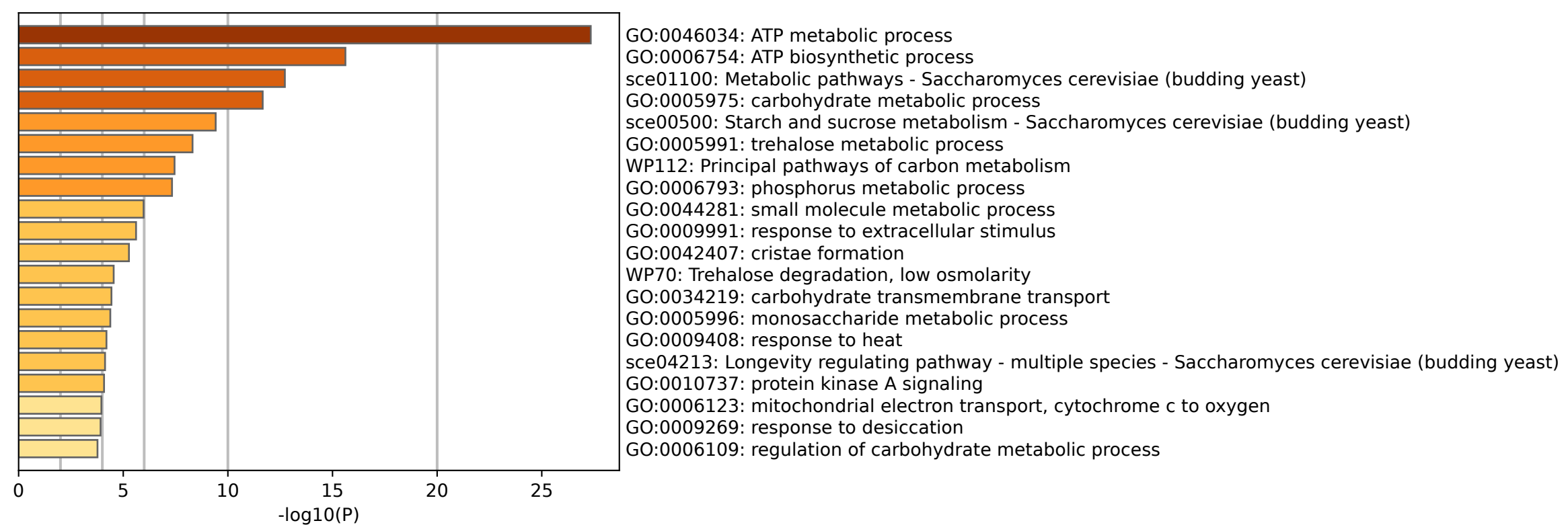

Supplement: Figure 2—source data 1. [file elife-92178-fig2-data1.zip › RNA seq, TF and Metascape/Metascape analysis/Enrichment_heatmap/HeatmapSelectedGO.pdf]

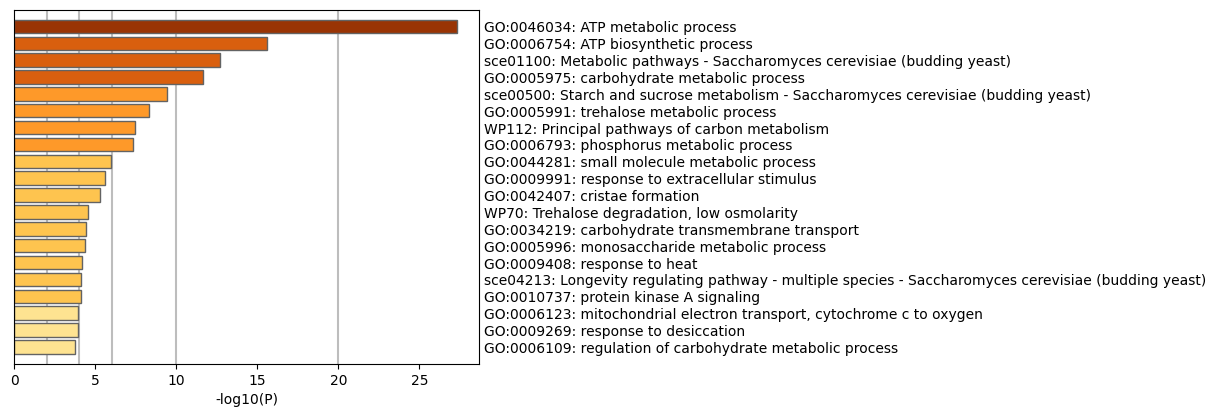

Supplement: Figure 2—source data 1. [file elife-92178-fig2-data1.zip › RNA seq, TF and Metascape/Metascape analysis/Enrichment_heatmap/HeatmapSelectedGO.png]

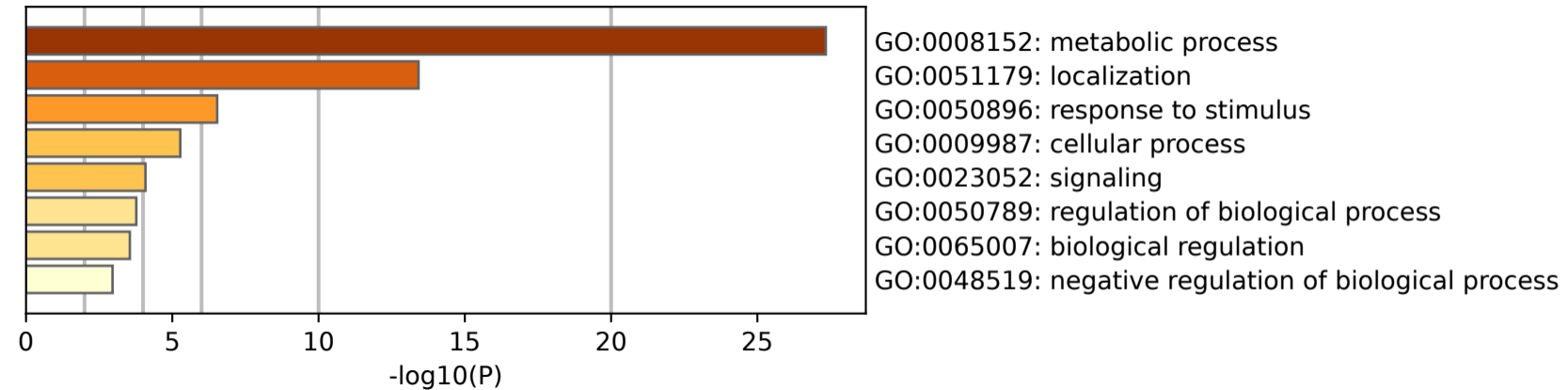

Supplement: Figure 2—source data 1. [file elife-92178-fig2-data1.zip › RNA seq, TF and Metascape/Metascape analysis/Enrichment_heatmap/HeatmapSelectedGOParent.pdf]

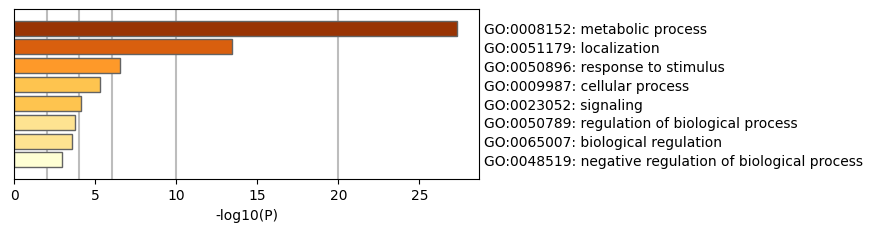

Supplement: Figure 2—source data 1. [file elife-92178-fig2-data1.zip › RNA seq, TF and Metascape/Metascape analysis/Enrichment_heatmap/HeatmapSelectedGOParent.png]

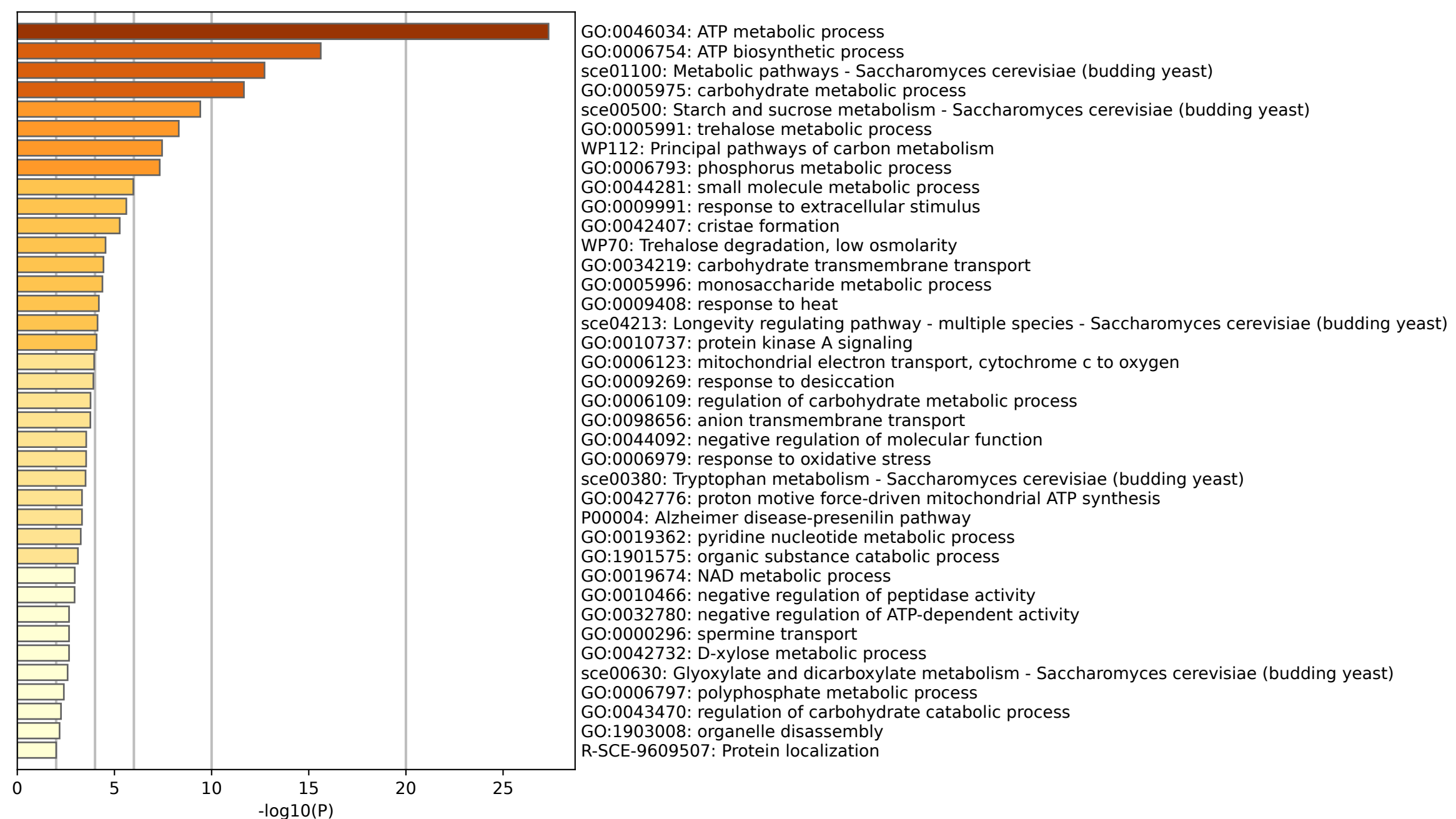

Supplement: Figure 2—source data 1. [file elife-92178-fig2-data1.zip › RNA seq, TF and Metascape/Metascape analysis/Enrichment_heatmap/HeatmapSelectedGOTop100.pdf]

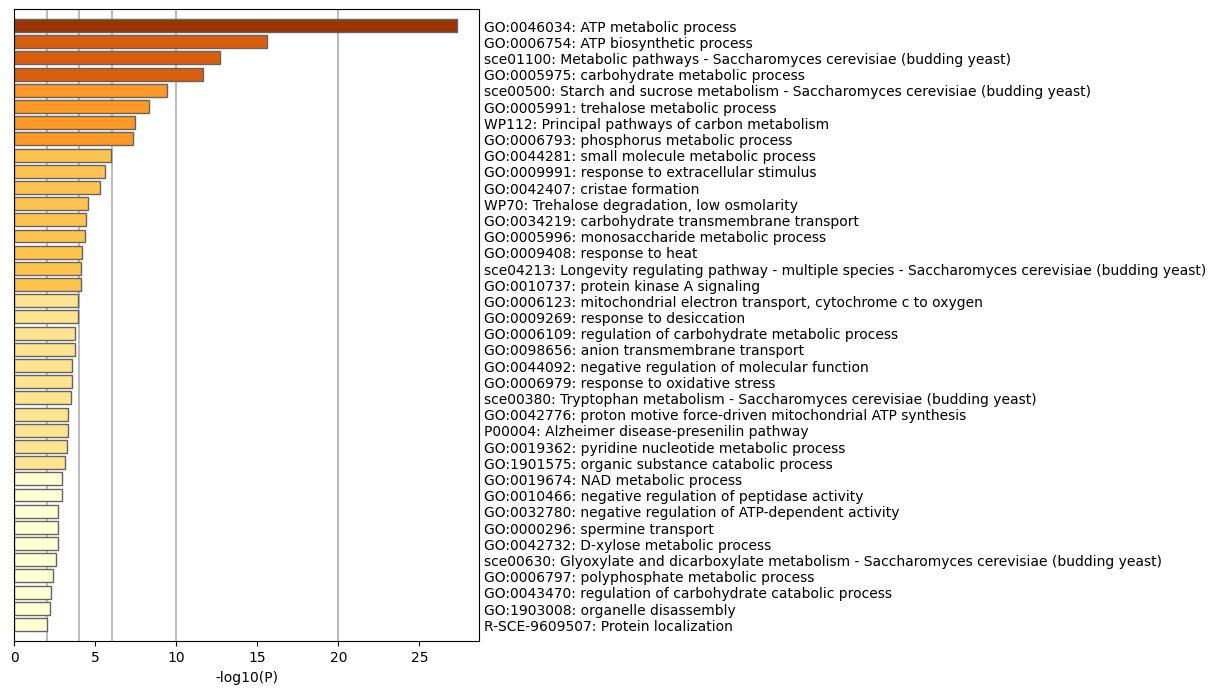

Supplement: Figure 2—source data 1. [file elife-92178-fig2-data1.zip › RNA seq, TF and Metascape/Metascape analysis/Enrichment_heatmap/HeatmapSelectedGOTop100.png]

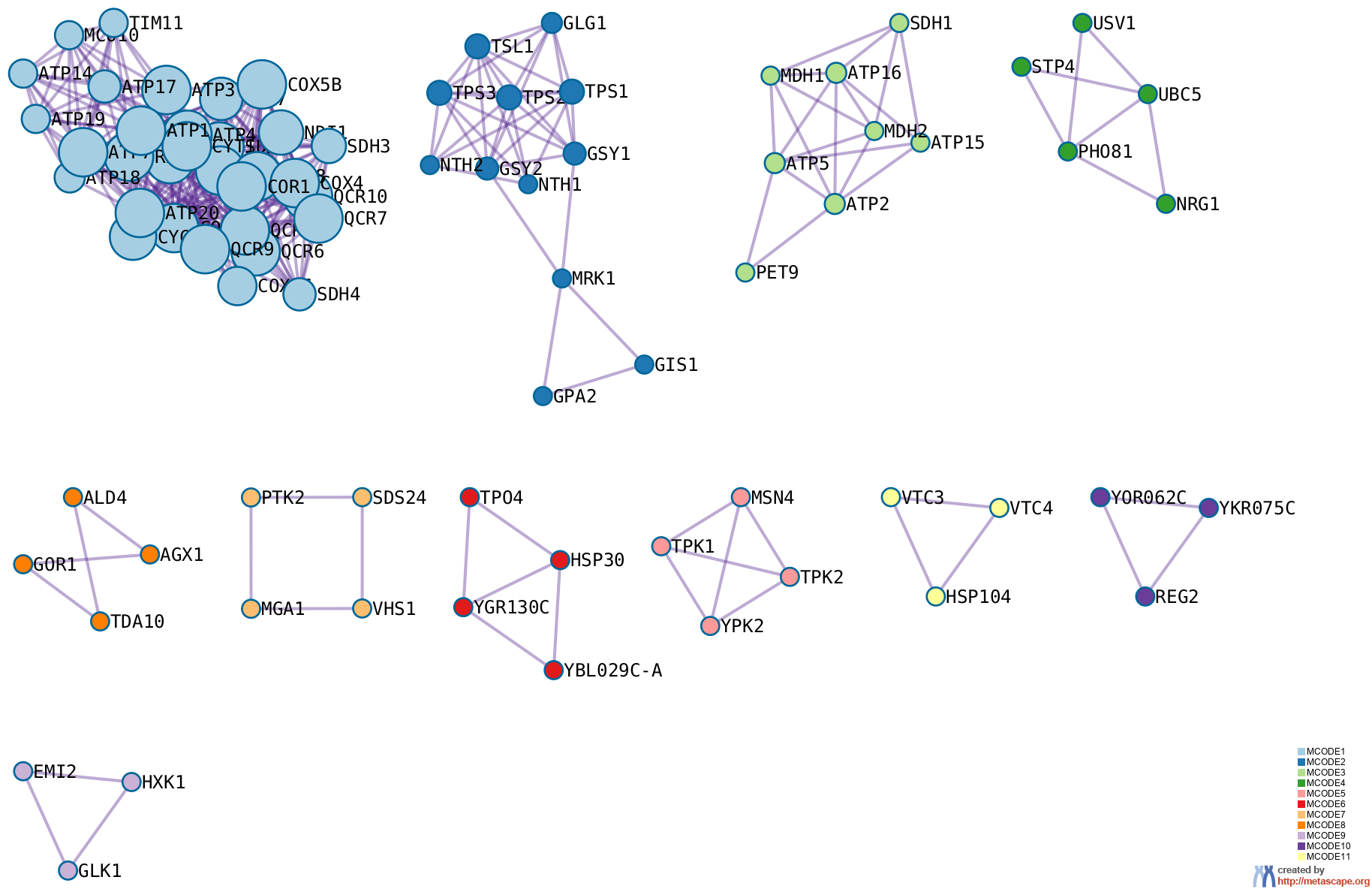

Supplement: Figure 2—source data 1. [file elife-92178-fig2-data1.zip › RNA seq, TF and Metascape/Metascape analysis/Enrichment_PPI/MyList_MCODE_ALL_PPIColorByCluster.png]

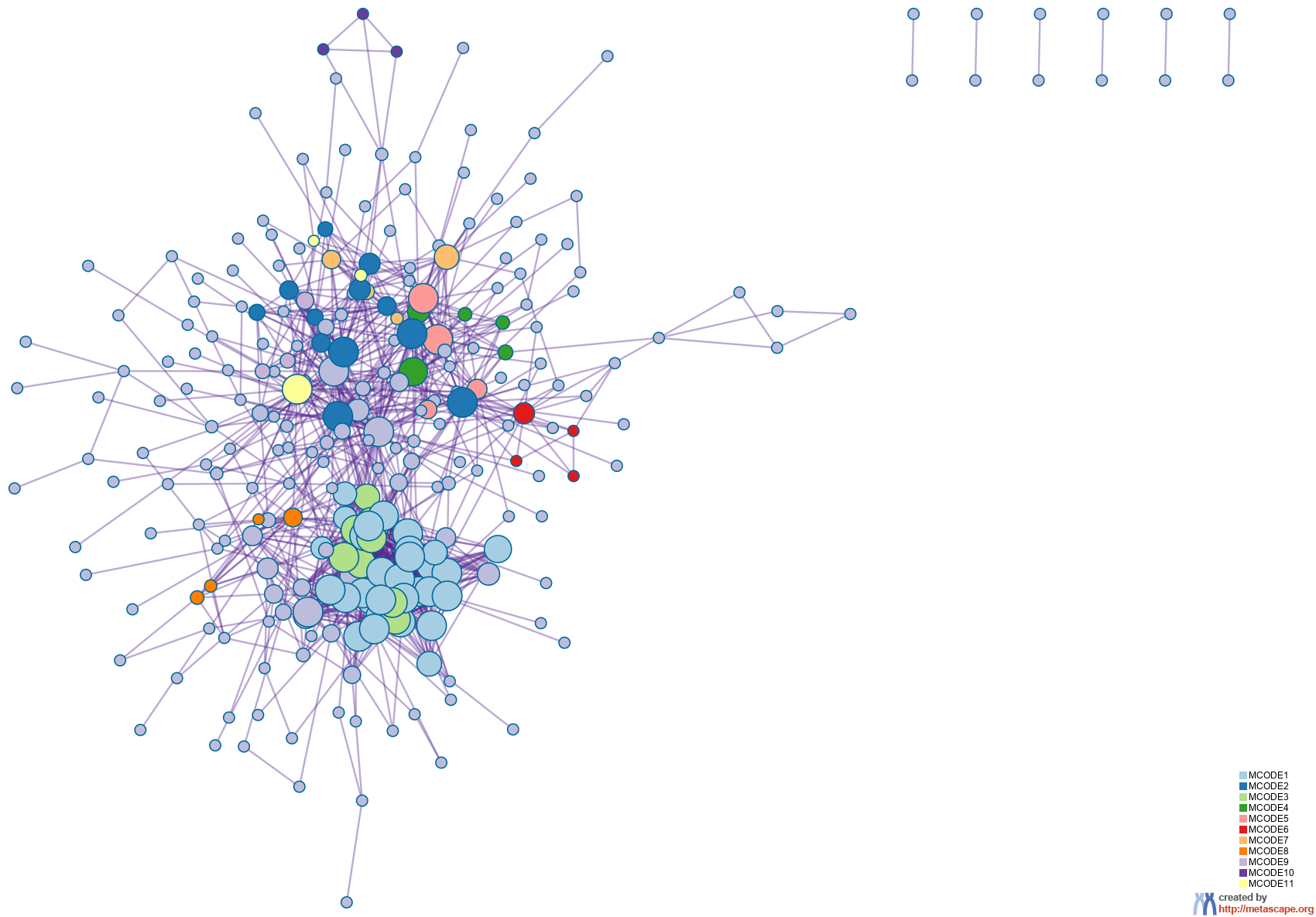

Supplement: Figure 2—source data 1. [file elife-92178-fig2-data1.zip › RNA seq, TF and Metascape/Metascape analysis/Enrichment_PPI/MyList_PPIColorByCluster.png]

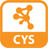

Supplement: Figure 2—source data 1. [file elife-92178-fig2-data1.zip › RNA seq, TF and Metascape/Metascape analysis/icon/CYS48.png]

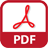

Supplement: Figure 2—source data 1. [file elife-92178-fig2-data1.zip › RNA seq, TF and Metascape/Metascape analysis/icon/PDF48.png]

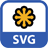

Supplement: Figure 2—source data 1. [file elife-92178-fig2-data1.zip › RNA seq, TF and Metascape/Metascape analysis/icon/SVG48.png]

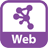

Supplement: Figure 2—source data 1. [file elife-92178-fig2-data1.zip › RNA seq, TF and Metascape/Metascape analysis/icon/WEB_CYS48.png]
